# Loss of *ALDH18A1* function is associated with a cellular lipid droplet phenotype suggesting a link between autosomal recessive cutis laxa type 3A and Warburg Micro syndrome

**DOI:** 10.1002/mgg3.70

**Published:** 2014-03-11

**Authors:** Mark T Handley, André Mégarbané, Alison M Meynert, Stephen Brown, Elisabeth Freyer, Martin S Taylor, Ian J Jackson, Irene A Aligianis

**Affiliations:** 1 MRC Human Genetics Unit, Institute of Genetics and Molecular Medicine, University of EdinburghEdinburgh, UK; 2 Institut Médical Jérôme Lejeune et Fondation Jérome LejeuneParis, France; 3 Unité de Génétique Médicale, Faculté de Médecine, Université Saint-JosephBeirut, Lebanon

**Keywords:** ARCL3A, cutis laxa, lipid droplets, Warburg Micro syndrome

## Abstract

Autosomal recessive cutis laxa type 3A is caused by mutations in *ALDH18A1*, a gene encoding the mitochondrial enzyme Δ^1^-pyrroline-5-carboxylate synthase (P5CS). It is a rare disorder with only six pathogenic mutations and 10 affected individuals from five families previously described in the literature. Here we report the identification of novel compound heterozygous missense mutations in two affected siblings from a Lebanese family by whole-exome sequencing. The mutations alter a conserved C-terminal domain of the encoded protein and reduce protein stability as determined through Western blot analysis of patient fibroblasts. Patient fibroblasts exhibit a lipid droplet phenotype similar to that recently reported in Warburg Micro syndrome, a disorder with similar features but hitherto unrelated cellular etiology.

Syndromic cutis laxa (CL) comprises a group of distinct but clinically overlapping disorders within which inelastic wrinkled and redundant skin is a common feature (Mohamed et al. 2011). It is genetically heterogeneous and can be categorized according to autosomal dominant, recessive, or X-linked modes of inheritance. Within the category of autosomal recessive CL, Type I (ARCL1, MIM 219100, 614437, 613177) is associated with pulmonary emphysema and poor prognosis. It is caused by mutations in *ELN* (MIM 130160), *FBLN5* (MIM 604580), *EFEMP2* (MIM 604633), or *LTBP4* (MIM 604710) which encode secreted proteins that interact with the extracellular matrix (ECM)(Elahi et al. 2006; Hucthagowder et al. 2006; Megarbane et al. 2009; Urban et al. 2009). Type II (ARCL2, MIM 219200, 612940) is associated with developmental delay, skeletal abnormalities and characteristic facies. ARCL2 can be caused by mutations in *ATP6V0A2* (MIM 611716) which encodes a component of a Golgi-localized H^+^-ATPase (Kornak et al. 2008). A related disorder, geroderma osteodysplasticum (GO, MIM 231070) is caused by mutations in *GORAB* (MIM 607983), which encodes a RAB6-interacting Golgin (Hennies et al. 2008). Type III ARCL, also known as De Barsy syndrome, is associated with cognitive impairment and motor deficits in addition to developmental delay and abnormal facies. ARCL3A (MIM 219150) is caused by mutations in *ALDH18A1* (MIM 138250) and ARCL3B (MIM 614438) is caused by mutations in *PYCR1* (MIM 179035). These genes encode mitochondrial enzymes involved in proline synthesis, Δ^1^-pyrroline-5-carboxylate synthase (P5CS) and pyrroline-5-carboxylate reductase 1, respectively (Baumgartner et al. 2000; Reversade et al. 2009). Mutations in *PYCR1* are a more frequently reported cause of ARCL3 than mutations in *ALDH18A1*. Additionally, reflecting clinical overlap between conditions and possibly also a variable clinical presentation, *PYCR1* mutations have been described as causing ARCL2B (MIM 612940) and GO as well as ARCL3B (Guernsey et al. 2009; Yildirim et al. 2011; Dimopoulou et al. 2013; Scherrer et al. 2013).

Variable features of ARCL3 resulting from *ALDH18A1* mutations include microcephaly and cataracts. Microcephaly has been reported in 7/10 cases, a similar proportion to that seen in patients with *PYCR1* mutations. In contrast, bilateral cataracts have been seen in 6/10 cases and so appear more frequent than in patients with *PYCR1* mutations, only ∼10% of whom are affected (Zampatti et al. 2012; Dimopoulou et al. 2013). Microcephaly and cataracts in particular, and also symptoms of developmental delay, hypotonia with ascending spastic paraplegia and contractures, and hypogenesis of the corpus callosum represent an overlap in phenotype between ARCL3A and Warburg Micro syndrome (WARBM, MIM 600118, 614225, 614222)(Handley et al. 2013)(see Table 1). In this study, we show that the overlapping features of ARCL3A and WARBM also extend to the cellular level, with fibroblasts derived from an ARCL3A patient displaying a lipid droplet (LD) phenotype very similar to that recently reported in WARBM (Liegel et al. 2013).

**Table 1:** Comparison of the phenotypic features of ARCL3 and WARBM

Condition	ARCL3A *(ALDH18A1)*	ARCL3B *(PYCR1)*	Warburg Micro syndrome *(RAB3GAP1/RAB3GAP2/RAB18/TBC1D20)*
Postnatal growth retardation	+	+	+
Postnatal microcephaly	++	++	++
Cognitive impairment	++	+	++
Hypotonia	++	++	++
Athetoid movements	+	+	+
Contractures	+	+	+
Neuropathy	+	−	+
Corpus callosum dysgenesis	+/−	+/−	++
Cataracts	+	+/−	++
Microphthalmia	−	−	+
Microcornea	−	−	+
Hypogonadism	+/−	−	+
Cutis laxa	+	++	−

‘++’ indicates a prominent manifestation of the syndrome whereas ‘+’ indicates a commonly encountered trait and ‘−’ a rarely encountered trait.

The patients in this study are the only two boys born to healthy nonrelated Lebanese parents. Patient 1 was first seen when he was 18 months old. At birth, his weight was 2700 g, and his length 48.5 cm. According to the parents, he was quiet, hypotonic, had few episodes of constipation that required glycerin suppositories, and redundant skin that disappeared around the age of 1 year. At 7 months, he was diagnosed to have bilateral cataracts that were removed surgically. Upon examination, his weight was 8.1 kg (3rd percentile), height 77.5 cm (5th percentile), and head circumference 42.3 cm (<3rd percentile). He had a small and receding forehead, a flat occiput, deeply set eyes, cupped, simple, large ears, a hooked nose with a prominent columella, short philtrum, long, thin fingers with some ulnar deviation. External genitalia showed bilateral ascended testes. Neurological examination revealed a hypotonic boy, unable to support himself in sitting, decreased muscle bulk, and increased deep tendon reflexes. Ophthalmological examination revealed the presence of a bilateral aphakia. Abdominal ultrasonography and echocardiography did not show any abnormalities. A brain MRI was performed and revealed thinning of the corpus callosum with enlargement of the subarachnoid space anteriorly within the hemispheric fissure, lateral and 3rd ventricles were prominent in size without evidence for hydrocephaly, cerebellar hypoplasia, mildly delayed myelination, a mega cisterna magna, small optic chiasm, and abnormal lenses within the globes. Auditory evoked potential was normal. A total body x-ray revealed a short left first metacarpal, and a bilateral coxa valga. Upon several examinations, the clinical course was unremarkable except that he had severe developmental delay. At the last follow-up, he was 11 years old. He could not sit unaided and was speechless. Head circumference was 43 cm, height 104 cm, and weight 12 kg (all values largely below the third percentile). In addition, to the known features, he had deep set eyes, prominent cheeks, hypoplastic alae nasi, very thin skin with visible fine vessels, and multiple joint contractures.

Patient 2 was first evaluated by us at the age of 1 year. At birth, his weight was 2600 g, and his length 49 cm. His clinical course was identical to that of his affected brother except that he did not have cataracts, and had a normal skin appearance at birth. At examination, he was 5 years and 9 months. His weight was 11 kg, height 92.5 cm, and head circumference 39.7 cm (<3rd percentile). Physical examination showed the same dysmorphic features present in his brother with the addition of camptodactyly to the left fingers. Abdominal ultrasonography did not show any abnormalities. Metabolic screening was carried out for both patients and no anomalies found. Serum and urine amino acids, urine organic acids, very long chain fatty acid (VLCFA), phytanic acid, electrolytes, aspartate aminotransferase (AST), alanine aminotransferase (ALT), creatine kinase (CK), carnitine, acylcarnitine, acylglycine and tests for congenital disorders of glycosylation were all normal.

In order to identify the genetic basis for the disease, whole-exome sequencing was carried out using DNA from patient 1. A total of 51,366 exonic variants were identified and previously reported variants were filtered according to functional effect and frequency data from the Exome variant server and 1000 genomes project (see Data S1). Synonymous variants and variants with a frequency >0.002 in the general population were excluded from analysis. Among the remaining 441 rare variants, we identified two novel missense mutations in *ALDH18A1*, c.2246G>A, p.Arg749Gln and c.2294G>A, p.Arg765Gln. These mutations were confirmed by Sanger sequencing and were present in both affected siblings. The c.2246G>A, p.Arg749Gln mutation was transmitted by the father and the c.2294G>A, p.Arg765Gln mutation by the mother (Fig. 1A). Neither mutation was identified in >400 control chromosomes. Because of the clinical overlap between ARCL3A and WARBM, we carried out screening for *ALDH18A1* mutations in an additional 51 families with a WARBM phenotype but in whom there were no identified mutations in the known disease genes, *RAB3GAP1*, *RAB3GAP2*, *RAB18*, or *TBC1D20*. This cohort is detailed in Handley et al. (2013). No further likely pathogenic mutations were identified and the coding variants c.896C>T, p.Thr299Ile (rs2275272), and c.1115C>A, p.Ser372Tyr (rs3765571), were seen at a frequency consistent with that in the general population (10/50 and 3/50 respectively).

**Figure 1:**
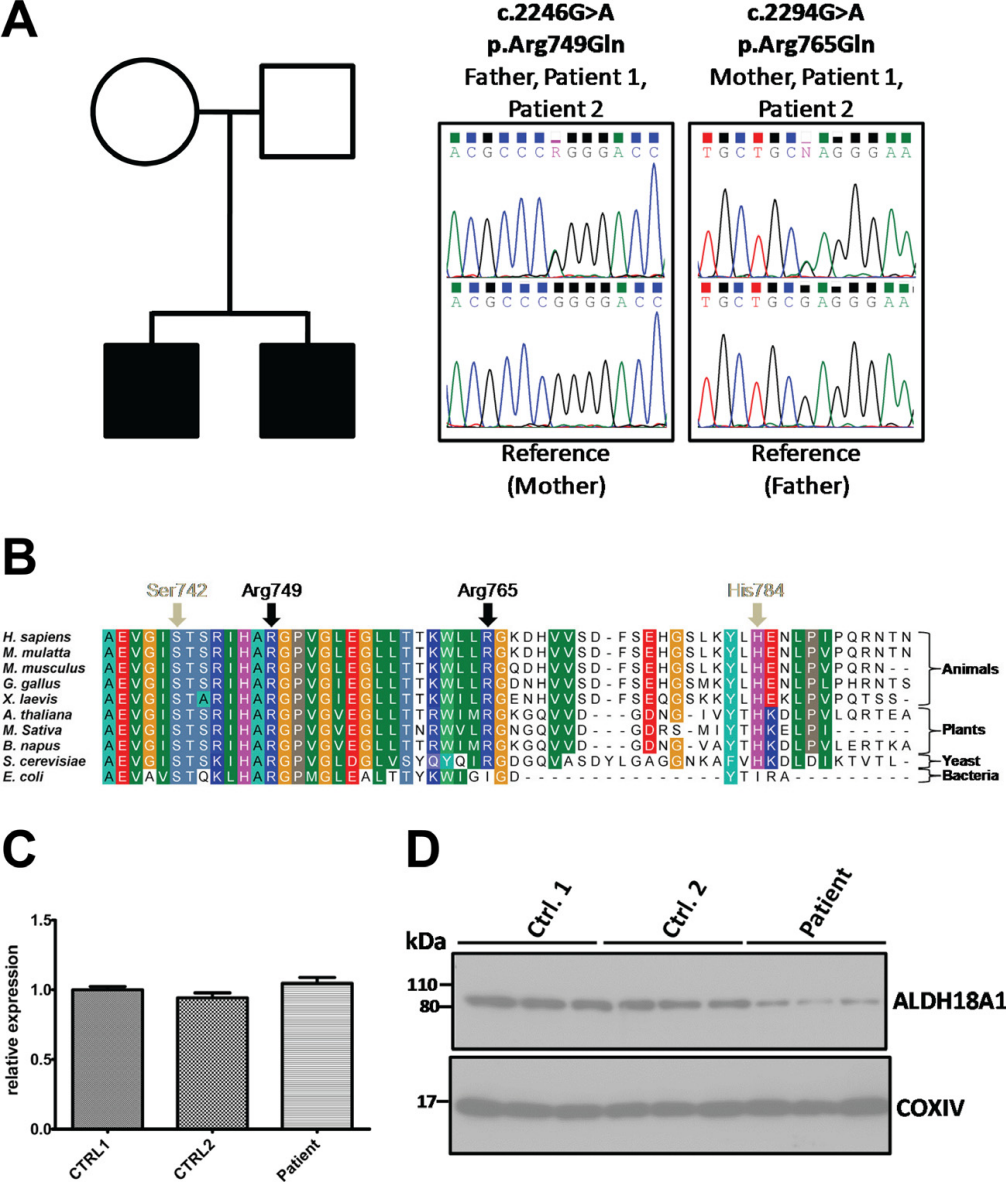
Novel compound heterozygous mutations in *ALDH18A1* cause ARCL3A. (A) Two affected children from a Lebanese family carry both a paternally inherited c.2246G>A, and a maternally inherited c.2294G>A mutation in *ALDH18A1*. Patient (upper) and reference (lower) electropherograms show these sequence changes, which lead to nonsynonymous p.Arg749Gln and p.Arg765Gln mutations in the encoded ALDH18A1 protein. Mutations were confirmed by direct sequencing. Nucleotide numbering reflects cDNA numbering with +1 corresponding to the A of the ATG translation initiation codon in the reference sequence NM_002860.3. Peptide residue numbering reflects protein primary structure with p.Met1 corresponding to the first methionine in the reference sequence NP_002851.2. (B) Multiple sequence alignment of ALDH18A1 ortholouges from various species shows that Arg749 and Arg765 are located in a highly conserved C-terminal domain of the protein close to the site of other residues reported as mutated in ARCL3A, Ser742 (Zampatti et al. 2012), and His784 (Bicknell et al. 2008). The alignment was generated using ClustalW software (Thompson et al. 1994). (C) Quantitative RT-PCR shows that expression of the ALDH18A1 transcript is similar in control and patient-derived fibroblasts. Data shown are derived from analysis of 6 cDNAs per genotype, each analysed in triplicate. Primers were designed using the Universal ProbeLibrary Assay Design Center (Roche, Welwyn Garden City, UK) and are listed in supplemental methods (Table S1). Error bars represent SEM. (D) Western blotting shows that levels of ALDH18A1 protein are reduced in patient-derived fibroblasts. Blotting for COXIV serves as a control. Each lane on the blots shown corresponds to an individual lysate sample, and each blot is representative of at least two independent experiments.

Both of the identified *ALDH18A1* mutations are in the last coding exon of the gene and affect a highly conserved C-terminal domain of the encoded protein in which pathogenic mutations have been described previously (Bicknell et al. 2008; Zampatti et al. 2012) (see Fig. 1B). Furthermore, software tools including Mutation Taster and PolyPhen-2 indicated that they were likely to be damaging. Therefore, in order to determine the consequence of the mutations on mRNA and protein expression, we carried out qPCR and Western blot analysis on fibroblasts derived from one of the patients and two control individuals. We found that levels of ALDH18A1 mRNA were comparable between patient and control lines (Fig. 1C); but that levels of ALDH18A1 protein were clearly reduced in the patient cells (Fig. 1D). Reduced ALDH18A1/PYCR1 protein expression has previously been reported in ARCL3 (Baumgartner et al. 2000; Martinelli et al. 2012). Therefore, taken together, these data indicate that the identified *ALDH18A1* mutations are pathogenic and that one or both of them has an effect on protein stability.

The mechanism for ARCL3A disease pathogenesis is not yet clear. Initial reports showed that a c.251G>A mutation leading to a p.Arg84Gln substitution in the conserved *γ*-glutamyl kinase domain of ALDH18A1 are associated with impaired enzymatic activity, and reduced ornithine, citrulline, arginine, and proline and paradoxical hyperammonemia in patients (Baumgartner et al. 2000, 2005). Furthermore, similar findings have been made in a patient carrying a heterozygous *de novo* c.277G>A mutation leading to a p.Gly93Arg substitution (Martinelli et al. 2012). However, these metabolic features have not been observed in patients carrying other mutations (Bicknell et al. 2008; Skidmore et al. 2011; Zampatti et al. 2012). *In vitro* analysis of patient fibroblasts carrying a c.2350C>T, p.His784Tyr mutation in a domain of the protein thought to be involved in oligomerization, for example, show normal proline synthesis (Bicknell et al. 2008). These data suggest that nonsynonymous mutations or splicing mutations may differentially affect protein expression or function while at the same time contributing to an otherwise broadly similar disease phenotype.

Both ALDH18A1 and PYCR1 are mitochondrial proteins, and mutations in *PYCR1* have been linked to mitochondrial dysfunction and increased susceptibility to oxidative stress (Reversade et al. 2009). However, corresponding experiments on cells deficient in ALDH18A1 have not identified a similar cellular phenotype (Martinelli et al. 2012). Accordingly, labeling of ALDH18A1(p.Arg749Gln/Arg765Gln) fibroblasts with the mitochondrial marker Mitotracker red CMXRos (Life technologies, Paisley, UK) and an antibody to the mitochondrial protein COXIV (New England Biolabs, Hitchin, UK) did not identify any gross morphological abnormalities or defects in the filamentous mitochondrial network as compared to controls (Fig. S1A). Furthermore, there was no differential effect of H_2_O_2_-induced oxidative stress on these cells (Fig. S1B). We extended our investigation to include other intracellular compartments, but identified no alterations in endoplasmic reticulum (ER) labeled with an antibody against protein disulfide isomerise (PDI), *cis*-Golgi labeled with an antibody against GM130, trans-Golgi labeled with an antibody against golgin-97 or endosomes labeled with an antibody against EEA1 (Fig. S2).

In a recent study, WARBM has been linked to a cellular deficit in LD formation. Not only in human fibroblasts deficient in *RAB3GAP1*, *RAB18*, or *TBC1D20*, but also in mouse embryonic fibroblasts (mEFs) deficient in *Tbc1d20*, there is a significant increase in LD size when cells are treated with oleate for 18 or 24 h to induce LD formation (Liegel et al. 2013). We hypothesized that the similarities in the clinical presentation of WARBM and ARCL3A might be reflected at the cellular level and so characterized LD formation in the ALDH18A1(p.Arg749Gln/Arg765Gln) cells. As shown in Figure 2A and B, we loaded cells with 400 *μ*mol/L oleate complexed to BSA in full media for 6, 18, and 24 h and then fixed the cells and stained them with the neutral lipid stain BODIPY. Confocal microscopy with subsequent analysis using the thresholding and ‘analyse particles’ tools on imageJ showed that LDs are enlarged in ALDH18A1(p.Arg749Gln/Arg765Gln) cells as compared to two control fibroblast cell lines following both 18 and 24 h oleate treatment (Fig. 2B). The uptake of lipid by individual cells is highly variable. We therefore carried out additional experiments in which cells were again loaded with oleate but this time labeled with the vital LD stain BODIPY 558/568 C12 (Life Technologies) (Wang et al. 2010). Lipid uptake of individual cells was then quantified by FACS analysis and confirmed that this is increased in ALDH18A1(p.Arg749Gln/Arg765Gln) cells (Fig. 2C). Because the observed LD phenotype strongly resembled that of the WARBM cell lines, we carried out Western blotting for the WARBM gene products RAB3GAP1, RAB3GAP2, RAB18, and TBC1D20. However, there was no change in the levels of these proteins in ALDH18A1(p.Arg749Gln/Arg765Gln) cells as compared to the control lines. These data suggest that the LD phenotype in the ALDH18A1(p.Arg749Gln/Arg765Gln) cells is the result of a deficiency in a parallel pathway leading to aberrant LD formation rather than in a pathway that overlaps that involving the WARBM proteins at a molecular level.

**Figure 2:**
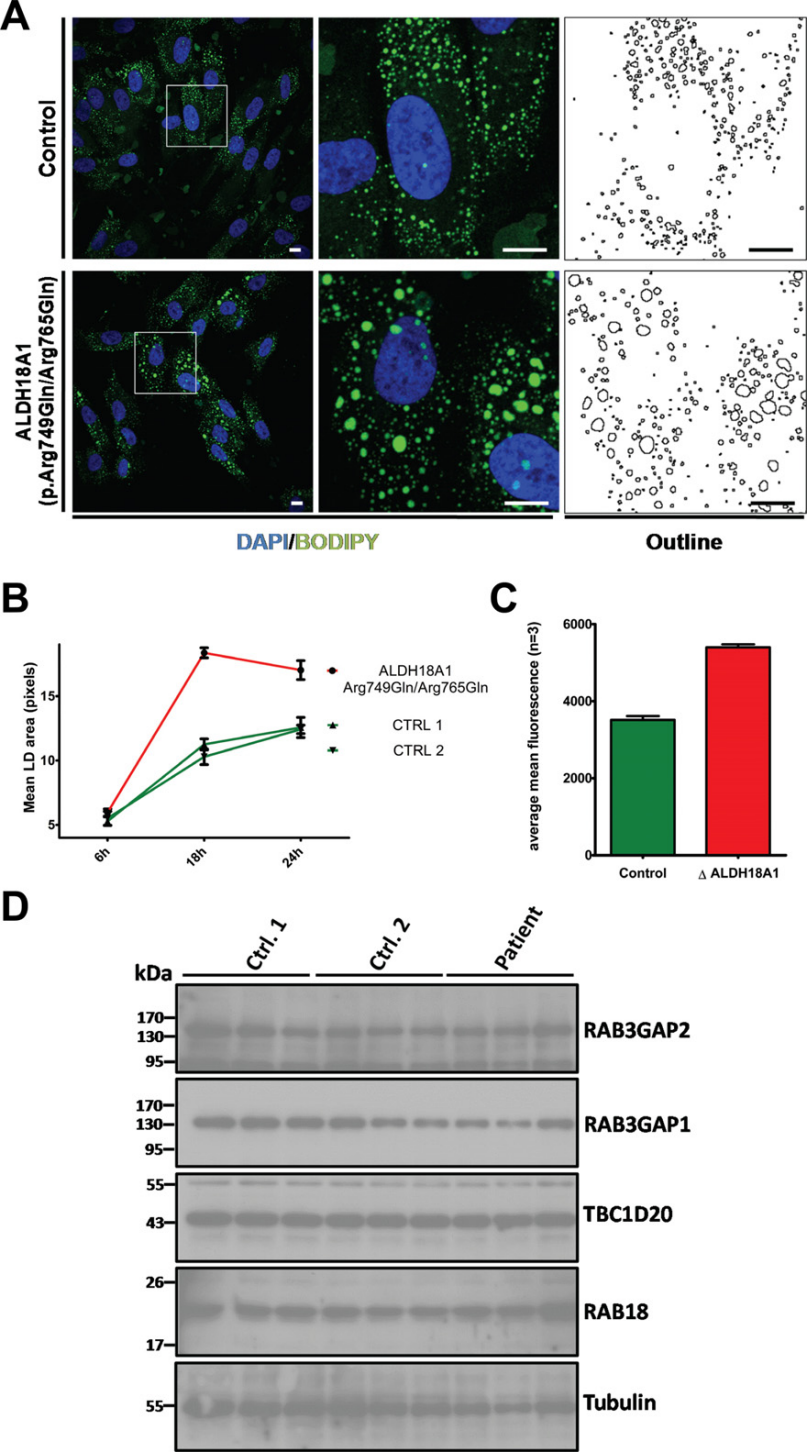
A lipid droplet (LD) phenotype in *ALDH18A1*-mutant fibroblasts. (A) Treatment of ALDH18A1(p.Arg749Gln/Arg765Gln) fibroblasts for 24 h with 400 *μ*mol/L oleate and subsequent staining of LDs with BODIPY493/503 (green) revealed a greater size of LDs when compared to controls. DNA was stained with DAPI (blue). Scale bars = 10 *μ*m. (B) Quantification of LD size in ALDH18A1(p.Arg749Gln/Arg765Gln) fibroblasts and two control fibroblasts lines following 6 h 18 h and 24 h treatment with 400 *μ*mol/L oleate. Data shown are derived from five frames/condition using the ‘analyse particles’ tool on imageJ and are representative of three independent experiments. (C) Quantification of lipid uptake by FACS. Control and ALDH18A1(p.Arg749Gln/Arg765Gln) fibroblasts were treated for 18 h with 400 *μ*mol/L oleate labeled with BODIPY 558/568 C12 and the fluorescence intensity of individual cells measured by FACS. Data shown correspond to average fluorescence from >3 replicate samples/genotype and are representative of at least two independent experiments. (D) Western blotting shows that levels of RAB3GAP2, RAB3GAP1, TBC1D20, and RAB18 proteins are unchanged in patient-derived fibroblasts as compared to controls. Blotting for *β*-tubulin serves as a control. Each lane on the blots shown corresponds to an individual lysate sample, and each blot is representative of at least two independent experiments. Error bars represent SEM.

Together, the above data identify two novel pathogenic mutations in *ALDH18A1* as causative in ARCL3A and aberrant LD formation as a cellular phenotype associated with this disease. The mechanism by which mitochondrial ALDH18A1 dysfunction affects LDs is unclear, though a working hypothesis is that the metabolic deficiency resulting from ALDH18A1-loss might disrupt cellular homeostasis. This might result in changes in LDs as there are many ways in which LDs and mitochondria are linked functionally. For example, LDs store cellular cholesterol esters and triglycerides whereas the initiation of steroid synthesis from cholesterol and fatty acid oxidation following lipolysis occurs in mitochondria (Walther and Farese 2012). It is also unclear, as yet, whether ARCL3A pathology is directly linked to LD dysfunction or whether both are linked to an underlying deficit. However, it is intriguing that LD dysfunction appears to be a cellular correlate for the similar phenotypes associated with multiple inherited diseases. LD defects have not only been found associated with WARBM, but also in several other similar syndromes. For example, mutations in *SPG20/Spartin* (MIM 607111) are associated with Troyer syndrome, a complex form of hereditary spastic paraplegia (MIM 275900) characterized by short stature and cognitive deficits in addition to progressive ascending spasticity. SPG20 has been found to associate with both LDs and mitochondria, and SPG20 deficiency has been linked to LD enlargement and accumulation (Lu et al. 2006; Eastman et al. 2009). Future work should seek to establish the extent to which this phenotype is indicative of a common pathology. In particular, it would be interesting to determine whether the LD dysregulation is a feature of ARCL3A resulting from other mutations in *ALDH18A1*, or indeed, ARCL resulting from mutations in other genes.

## Supporting Information

**Figure S1.** Morphologically normal mitochondria in *ALDH18A1*-mutant fibroblasts. **Figure S2.** Morphologically normal membrane compartments in *ALDH18A1*-mutatnt fibroblasts. **Table S1.** Primer sequences. **Data S1.** Materials and methods.
