# Supplementary material for: Loss of ALDH18A1 function is associated with a cellular lipid droplet phenotype suggesting a link between autosomal recessive cutis laxa type 3A and Warburg Micro syndrome
Source: Mol Genet Genomic Med. 2014 Mar 11;2(4):319–25. doi: 10.1002/mgg3.70 (PMC4113272; doi:10.1002/mgg3.70)
Supplement: Supplementary file 1 [file mgg30002-0319-SD1.docx]

**Supplementary material**

**Loss of *ALDH18A1* function is associated with a cellular lipid droplet phenotype suggesting a link between autosomal recessive cutis laxa type 3A and Warburg Micro syndrome.**

Mark T. Handley ^1^, André Mégarbané ^2^, Alison Meynert^1^, Stephen Brown ^1^, Elisabeth Freyer^1^, Martin Taylor^1^, Ian J. Jackson ^1^ and Irene A. Aligianis ^1^

1. MRC Human Genetics Unit, Institute of Genetics and Molecular Medicine, University of Edinburgh, Edinburgh, Scotland, UK
2. Institut Médical Jérôme Lejeune et Fondation Jérome Lejeune, Paris, France, Unité de Génétique Médicale, Faculté de Médecine, Université Saint-Joseph, Beirut, Lebanon

**Supplementary Figure 1.**

**Supplementary figure 1. Morphologically normal mitochondria in *ALDH18A1*-mutant fibroblasts.** (A) The filamentous mitochondrial network in control and patient-derived fibroblasts was labelled by incubation of live cells with 200nM Mitotracker CMXRos (red) for 30min followed by fixation and immunocytochemistry with an antibody to COXIV (green). DNA was stained with DAPI (blue). (B) Mitochondria in control and patient-derived fibroblasts were labelled with 200nM Mitotracker CMXRos (red) for 30min and then subjected to treatment with 200µM (top panels) or 500µM (bottom panels) H_2_O_2_. DNA was stained with Hoescht 33342. Treatment with 200µM H_2_O_2_ for 10mins had little apparent effect on Mitotracker staining in either cell line whereas treatment with 500µM H_2_O_2_ produced an increase in cytosolic mitotracker fluorescence accompanied by some indication of morphological change in the mitochondria. Scale bars = 10 µm.

**Supplementary figure 2. Morphologically normal membrane compartments in *ALDH18A1*-mutatnt fibroblasts.** Immunofluorescence analysis with PDI as an ER marker (A-B), GM130 as a *cis*-Golgi marker (C-D), Golgin-97 as a *trans*-Golgi marker (E-F) and EEA1 as an endosomal marker (G-H) did not identify any difference between patient and control fibroblasts. Scale bars = 10 µm.

**Supplementary materials and methods**

Whole-exome sequencing

An indexed SureSelect XT sequencing library was prepared using Genomic DNA from patient 1. The sequencing library was enriched for exonic sequence using the Agilent SureSelect Human All Exon 50 Mb kit. Sequencing was performed on an Illumina HiSeq 2000 sequencer. Sequence reads were quality checked with FASTQC 0.9.1 and aligned to the hg19 human genome reference assembly with BWA 0.5.9 followed by Stampy 1.0.21. Duplicate reads were marked with Picard MarkDuplicates 1.79. Reads were re-aligned around indels and scores re-calibrated with GATK 2.2-8-gec077cd. BAM file sorting, merging, and indexing was performed by Samtools 0.1.16. SNPs and small indels were called with GATK. Variant consequences in Ensembl 68 transcripts were determined with SnpEff 3.1 using the GRCh37.68 database.

No sequence read quality issues were reported by FASTQC. After alignment to the reference human genome, 25% of the 102,409 unpaired reads and 33,452,826 read pairs were marked as duplicates. At Phred-scaled threshold of 60 for both variant and genotype quality, and a minimum read depth of 10X, 50254 SNPs and 1112 indels occurring in exons were called. When filtered for minor allele frequency (MAF) < 0.01 in both 1000 Genomes and ESP6500 data sets, this reduced to 12083 SNPs and 644 indels. When filtered for MAF<0.002, 441 rare variants remained.

Subjects and mutation screening

Our cohort consists of patients with a spectrum of Warburg Micro syndrome disorders including ‘typical’ Micro syndrome, Martsolf syndrome and several atypical cases as previously described (Handley, et al., 2013). These individuals do not carry mutations in the coding sequences of previously analyzed genes, specifically *RAB3GAP1*, *RAB3GAP2*, *TBC1D20* and *RAB18*. Informed consent was obtained from all participating families, and the studies were approved by the Scottish Multicentre Research Ethics Committee (04:MRE00/19).

Mutation screening of the complete coding region of the *ALDH18A1* gene was performed by direct sequence analysis in both directions using genomic DNA extracted from venous blood according to standard procedures. Primers with M13 tags (Supplementary table 1) were designed from the genomic sequences to flank all coding exons and include all intron-exon boundaries using ExonPrimer software. Sequencing data was analyzed using Mutation Surveyor, version 3.30 (SoftGenetics). NCBI reference sequence accessions for *ALDH18A1*, *RAB3GAP1*, *RAB3GAP2*, *TBC1D20* and *RAB18* are NM_002860.3, NM_012233.2, NM_012414.3, NM_144628.2 and NM_021252.4 respectively. Mutations are named according to standard nomenclature (see http://www.hgvs.org/mutnomen/).

Cell culture

Human fibroblasts were cultured in Dulbecco's Modified Eagle's Medium (Gibco) supplemented with 20% fetal calf serum and 1% penicillin/streptomycin. Cells were maintained under hypoxic conditions (3%O2, 5%CO2) at 37°C.

Antibodies and reagents

Polyclonal rabbit anti-ALDH18A1 was obtained from Sigma Aldrich. Monoclonal mouse anti-PDI and polyclonal goat anti-beta tubulin were obtained from Abcam. Monoclonal rabbit anti-EEA1 and anti-COXIV were obtained from New England Biolabs. Monoclonal mouse anti-GM130 was obtained from BD biosciences and monoclonal mouse anti-Golgin-97 from Transduction Laboratories. Secondary antibodies Alexa Fluor 488-conjugated donkey anti-mouse or anti-rabbit (Life Technologies) and Peroxidase-conjugated anti-mouse or anti-Rabbit (Amersham) or anti-Goat (Life Technologies) were used. Hoechst 33342 and Mitotracker CMXRos were obtained from Life Technologies and H_2_O_2_ was obtained from Sigma Aldrich.

For lipid-loading experiments, Oleic acid (Sigma Aldrich) was complexed to bovine serum albumin (Sigma Aldrich) at an approximate molar ratio of 8:1, with or without the addition of BODIPY 558/568 C12 (Life Technologies) and then combined with full media (see above) to a final concentration of 400µM prior to use.

qPCR analysis

Control and patient fibroblasts were seeded onto 6-well plates and grown until confluent. Each well was then trypsinised, the cells were washed, and then RNA was extracted using an RNeasy mini kit (Qiagen) according to manufacturer’s instructions. Purified RNA was used immediately as a template for production of cDNA using a First Strand cDNA Synthesis Kit for RT-PCR (AMV) (Roche). qPCR analysis of the resulting cDNAs (6 per genotype) was carried out on a LightCycler 480 (Roche) using primers designed to amplify specifically from ALDH18A1 transcript. PCR amplification was quantified through binding of specific mono color hydrolysis probes (Roche) and analyzed using LightCycler 480 software version 1.5.0 (SP4) (Roche). Primers were designed using the Universal ProbeLibrary Assay Design Center and are listed in Supplementary Table 1.

Western blotting

For Western blot analysis, cells were seeded on 6-well plates at a density of 5 x 10^5^ cells/well and allowed to grow for 48 hours. They were then lysed in a buffer containing 0.5% (v/v) Nonidet P-40 in a solution of 150mM NaCl, 10mM EDTA and 50mM Tris-HCl (pH=7.5) to which a protease inhibitor cocktail (Roche) was added. Following total protein quantification, samples were combined with a reducing loading buffer and subjected to SDS-PAGE and Western blotting carried out according to standard methods.

Western blots were first blocked in Tris-buffered saline (50mM Tris, 150 mM NaCl, pH 7.6) containing 4% powdered milk for a minimum of 1h and then probed with primary antibody in the same buffer, overnight at 4°C. Following washing, they were probed with an appropriate HRP-coupled secondary antibody for 1h before final washes. ECL 2 Western blotting substrate (Pierce) was used in combination with HyperFilm ECL (General Electric) to detect chemiluminescent signal and developed using a Konica Minolta SRX-101A.

Immunocytochemistry and imaging

For immunocytochemistry, cells were seeded onto glass coverslips in 24-well plates at a density of 1 x 10^5^ cells/well and allowed to adhere overnight, fixed for 30 minutes at room temperature in 4% (w/v) paraformaldehyde in phosphate-buffered saline (PBS) and then permeablized in PBS containing 10% (v/v) donkey serum (Sigma Aldrich) and 0.1% (vol/vol) Triton-X 100. Coverslips were probed with primary antibodies overnight at 4 °C and secondary antibodies were applied for 1h at room temperature.

For lipid-loading experiments, cells were seeded as above, then treated with 400µM oleate for 6, 18 or 24 hours to induce lipid droplet (LD) formation. Following fixation (as above) LDs were stained with a 1µg/ml solution of BODIPY 493/503 (Life Technologies) in 150mM NaCl for 10 minutes at room temperature. Coverslips were then mounted and stained with DAPI prior to imaging.

Cells were imaged on a Nikon A1R confocal microscope using a 60x oil immersion objective with a 1.4 numerical aperture. The pinhole was set to airy1. DAPI/Hoechst 33342 were excited using a 403.5nm laser, and emitted light was collected at 425-475nm. BODIPY/Alexa Fluor 488 were excited using a 488nm laser, and emitted light was collected at 500-550nm. Mitotracker CMXRos was excited using a 561.4nm laser, and emitted light was collected at 570-620nm.

FACS analysis

For FACS analysis, cells were seeded onto glass coverslips in 6-well plates at a density of 5 x 10^5^ cells/well and allowed to adhere overnight. For lipid-loading, coverslips were transferred to 10cm dishes with media containing 400µM oleate and 6µM BODIPY 558/568 C12 (Life Technologies) for 18 hours. Control and ALDH18A1(p.Arg749Gln/Arg765Gln) coverslips were combined in each dish and were therefore loaded under identical conditions. Following loading, cells on each coverslip were trypsinsed, washed twice with PBS, then resuspended in 100µl HBSS (Sigma) prior to analysis. Flow cytometry analysis of the cells was performed using a FACSAria2 SORP instrument (BD) equipped with 405nm, 488nm and 561nm lasers. Single cells were gated using forward scatter and side scatter and cells exhibiting high autofluorescence were excluded from analysis. BODIPY 558/568 C12 was detected using the 585/15BP filter. Data was analyzed using FACSDiva software (BD) Version6.1.3 and Flowjow (Tree Star Inc.) Version 7.6.4. Mean fluorescence intensity/cell was determined for each coverslip and the data shown are from >3 coverslips/genotype and are representative of at least two independent experiments.

**Supplementary table 1. Primer sequences.**

| ALDH18A1_ex1_F | GTAGCGCGACGGCCAGTCCAGCCTGTGTGACAAAATG |
| --- | --- |
| ALDH18A1_ex1_R | CAGGGCGCAGCGATGACCCGAGGGTAAGGGGAAGTAG |
| ALDH18A1_ex2_F | GTAGCGCGACGGCCAGTATGTCGTCTCTCACTGGCG |
| ALDH18A1_ex2_R | CAGGGCGCAGCGATGACCGCTAGATAGAGCTTTTGATACACTG |
| ALDH18A1_ex3_4_F | GTAGCGCGACGGCCAGTGCAAGTGATGAAGCTGACACC |
| ALDH18A1_ex3_4_R | CAGGGCGCAGCGATGACACTCGATAGCCCACCCTACC |
| ALDH18A1_ex5_F | GTAGCGCGACGGCCAGTAACTTTTCCATCTTGTTTATGCC |
| ALDH18A1_ex5_R | CAGGGCGCAGCGATGACCCTAAAGTTTAACTCCCCTGCTC |
| ALDH18A1_ex6_F | GTAGCGCGACGGCCAGTCCCTTTGTCAGAACATATGGC |
| ALDH18A1_ex6_R | CAGGGCGCAGCGATGACTAGCTTGGTTTTGCCTGAGC |
| ALDH18A1_ex7_F | GTAGCGCGACGGCCAGTGCCTACTATCTGAACCATTAACCC |
| ALDH18A1_ex7_R | CAGGGCGCAGCGATGACAAAGTACAGAGTGATTGGCAGG |
| ALDH18A1_ex8_F | GTAGCGCGACGGCCAGTCGATTTTGCAAATAGCGTTC |
| ALDH18A1_ex8_R | CAGGGCGCAGCGATGACGGTTGAGCTGGAAAGTGTGTG |
| ALDH18A1_ex9_F | GTAGCGCGACGGCCAGTGAATAAAGATGGAGTCAGCAGG |
| ALDH18A1_ex9_R | CAGGGCGCAGCGATGACTTGTGCATGTGCTGGTAGTTC |
| ALDH18A1_ex10_F | GTAGCGCGACGGCCAGTAGCCACCCACCTACCCTC |
| ALDH18A1_ex10_R | CAGGGCGCAGCGATGACGTGATGACCGTGGATGTGG |
| ALDH18A1_ex11_F | GTAGCGCGACGGCCAGTCTCCAATATCCACACCAGCC |
| ALDH18A1_ex11_R | CAGGGCGCAGCGATGACGGTTGGTAGCTGTAGCCTGC |
| ALDH18A1_ex12_F | GTAGCGCGACGGCCAGTTGCTGGAGTGTCAAGTCTGC |
| ALDH18A1_ex12_R | CAGGGCGCAGCGATGACGATGAAACAGCATAGGCCTTC |
| ALDH18A1_ex13_F | GTAGCGCGACGGCCAGTGGCAGCTGGATATTCACATTTAG |
| ALDH18A1_ex13_R | CAGGGCGCAGCGATGACTTTTCACAGTGTCTCACTTAGTTCTC |
| ALDH18A1_ex14_F | GTAGCGCGACGGCCAGTCACTTTACTTAGCATGGAACTTTG |
| ALDH18A1_ex14_R | CAGGGCGCAGCGATGACATGTATGTGGATTCCGAGGC |
| ALDH18A1_ex15_F | GTAGCGCGACGGCCAGTAATGCAACCACTACTAAACATTACC |
| ALDH18A1_ex15_R | CAGGGCGCAGCGATGACTTGGGCTGTGGTTTTACAGG |
| ALDH18A1_ex16_F | GTAGCGCGACGGCCAGTAAGGGGAGGTGTCTTTCCC |
| ALDH18A1_ex16_R | CAGGGCGCAGCGATGACAGACCCTGCCCTGATCTGTC |
| ALDH18A1_ex17_F | GTAGCGCGACGGCCAGTTTCCAACAGGCAGACCCTAC |
| ALDH18A1_ex17_R | CAGGGCGCAGCGATGACGTACCAAGTGAGGGGAAAGC |
|  |  |
| ALDH18A1_qPCR_F | TCTCGTCCTGACTGTCTACCC |
| ALDH18A1_qPCR_R | TAACAAGCCATTGCCACTTG |

**References**

Handley MT, Morris-Rosendahl DJ, Brown S, Macdonald F, Hardy C, Bem D, Carpanini SM, Borck G, Martorell L, Izzi C, Faravelli F, Accorsi P et al. 2013. Mutation spectrum in RAB3GAP1, RAB3GAP2, and RAB18 and genotype-phenotype correlations in warburg micro syndrome and Martsolf syndrome. Hum Mutat 34:686-96.

**URLs**

PolyPhen-2: <http://genetics.bwh.harvard.edu/pph2/index.shtml>

Mutation Taster: <http://doro.charite.de/MutationTaster/index.html>

ExonPrimer: http://ihg.gsf.de/ihg/ExonPrimer.html

FASTQC: http://www.bioinformatics.babraham.ac.uk/projects/fastqc/

Stampy: http://www.ncbi.nlm.nih.gov/pubmed/20980556

BWA: http://www.ncbi.nlm.nih.gov/pubmed/19451168

Picard: http://picard.sourceforge.net/

GATK: http://www.broadinstitute.org/gsa/wiki/index.php/Citing_the_GATK

Samtools: http://www.ncbi.nlm.nih.gov/pubmed/19505943

SnpEff: http://snpeff.sourceforge.net/faq.html#How_do_I_cite_your_work?

Ensembl: http://www.ncbi.nlm.nih.gov/pubmed/22086963

1000 Genomes: http://www.1000genomes.org/faq/how-do-i-cite-1000-genomes-project

ESP6500: http://evs.gs.washington.edu/EVS/ (see Data Usage and Release)
